# Nursing students’ professional values for reinforcing the professional identity

**DOI:** 10.1590/0034-7167-2022-0338

**Published:** 2023-10-06

**Authors:** Chennyfer Dobbins Abi Rached, Gleyciane Santos Vieira, Flávio Adriano Borges Melo, Mirian Cristina dos Santos Almeida, Vera Lúcia Mira, Herline Domercant, Nicole Yamada Crescente

**Affiliations:** I Universidade de São Paulo. São Paulo, São Paulo, Brazil; II Universidade Federal de São Carlos. São Carlos, São Paulo, Brazil; III Univesidade Federal do Tocantins. Palmas, Tocantins, Brazil

**Keywords:** Nursing Students, Code of Ethics, Nursing Ethics, Role of the Nursing Professional, Knowledge Management, Estudiantes de Enfermería, Códigos de Ética, Ética en Enfermería, Rol de la Enfermera, Gestión del Conocimiento, Estudantes de Enfermagem, Códigos de Ética, Ética em Enfermagem, Papel do Profissional de Enfermagem, Gestão do Conhecimento

## Abstract

**Objectives:**

to understand the nursing students’ professional values in different Brazilian universities and verify a correlation between the “Professional Value” and the sociodemographic variables.

**Methods:**

quantitative, cross-sectional, and descriptive study conducted through an electronic questionnaire with a Professional Values Scale (NPVS-3). Participants were Nursing students of all semesters from three universities - two in the Southeast region and one in the North region.

**Results:**

of the 337 participating Nursing students, 282 were female. The Caring dimension presented the highest score (mean=46.61), and Professionalism, the lowest score (mean=34.65). A statistically significant association was detected between the Caring dimension, “university where is attending,” and “gender.”

**Conclusions:**

the results indicate the Caring dimension as the one containing the most scored professional values since the nurses’ training, and the relation of those values in such dimension is more significant in the female sample.

## RESUMO

**Objetivos:**

conhecer os valores profissionais dos estudantes de Enfermagem em diferentes universidades brasileiras e verificar a existência de relação entre as variáveis “Valor profissional” e as variáveis sociodemográficas.

**Métodos:**

estudo quantitativo, transversal e descritivo, realizado por questionário eletrônico contendo a Escala de Valores Profissionais (NPVS-3). Participaram estudantes de todos os semestres de Enfermagem de três universidades - duas na Região Sudeste e uma na Região Norte.

**Resultados:**

dos 337 estudantes de Enfermagem participantes, 282 são do gênero feminino. A dimensão Cuidado apresentou maior pontuação (média=46,61); e o Profissionalismo, a menor pontuação (média=34,65). Encontrou-se associação estatisticamente significante da dimensão “Cuidado” com “universidade que estuda” e “gênero”.

**Conclusões:**

os resultados apontam a dimensão Cuidado como a que contém os valores profissionais mais pontuados desde a formação do enfermeiro, sendo que a relação desses valores em tal dimensão está mais acentuada na amostra do gênero feminino.

## RESUMEN

**Objetivos:**

conocer los valores profesionales de los estudiantes de Enfermería en diferentes universidades brasileñas y verificar la existencia de relación entre las variables “Valor profesional” y las variables sociodemográficas.

**Métodos:**

estudio cuantitativo, transversal y descriptivo, realizado por encuesta electrónica conteniendo la Escala de Valores Profesionales (NPVS-3). Participaron estudiantes de todos los semestres de Enfermería de tres universidades - dos en la Región Sudeste y una en la Región Norte.

**Resultados:**

de los 337 estudiantes de Enfermería participantes, 282 son del género femenino. La dimensión Cuidado presentó mayor puntuación (mediana=46,61); y el Profesionalismo, la menor puntuación (mediana=34,65). Encontrado relación estadísticamente significante de la dimensión “Cuidado” con “universidad que estudia” y “género”.

**Conclusiones:**

los resultados apuntan la dimensión Cuidado como la que contiene los valores profesionales más puntuados desde la formación del enfermero, siendo que la relación de esos valores en tal dimensión está más acentuada en la muestra del género femenino.

## INTRODUCTION

When researching the meaning of “human values,” the following definition can be found: “Moral principles that guide the conduct of people; those values constitute a set of rules established for a healthy coexistence within a society”^(1)^. Therefore, professional nursing values were developed as a model to reduce conflicts in decision-making, aiming to guide professional behavior. Professionals must consider personal values because they influence individual attitudes and behavior and may result in implications for the group^(1)^.

Professional values considered relevant for leadership skills are competence, commitment, mutual growth, and honesty. In that regard, nursing develops leadership due to the tasks required in its academic training and the institutional context, performing critical analysis, decision-making, team professionals’ allocation, and care implementation functions. Nursing leadership should, therefore, be considered based on care, which is the basis of the profession^(2)^. The presence of conflicting values between professionals and patients demands that the former be able to provide a care plan without harming their values, respecting honor, life, and individuality^(3-4)^. In other words, values are considered a universal code of orientation^(5)^.

According to Angela Susana Hernández Posada, in the article entitled *Algunas consideraciones acerca de los valores humanos y el profesional de enfermería* ^(3)^:

Understanding one’s personal and professional values and the ethical grounds structuring the nursing profession help nurses deal with the moral distress, uncertainty, challenges, and ethical dilemmas that increasingly influence nursing practice.

There is a statement in which professional values are not restricted to chronological time^(6)^. With the arrival of technology, questions emerge on the importance of values and reflections about the practice based on them. At this point, the Code of Ethics of Nursing Professionals (CEPE) is considered a reference for the professional values guiding the practice in Brazil^(7)^. CEPE gathers the fundamental principles for the conduct expected from nursing professionals; its subdivision includes rights, duties, prohibitions, infractions, and penalties. Its center is the individual, the family, and the collectivity. According to the Federal Council of Nursing (Cofen), nursing is responsible for citizens’ care, promotion, health rehabilitation, recovery, and disease prevention^(8)^.

This analysis aims to measure the professional values of nursing students to make educational planning more effective since the lack of their development during undergraduate studies generates significant impacts on attitudes and behaviors, leading to problems in leadership skills for the future professional career and failures in providing service.

## OBJECTIVES

To know the nursing students’ professional values in different Brazilian universities and verify the existence of an association between the “Professional Value” and the sociodemographic variables.

## METHODS

### Ethical aspects

All participants signed, in digital format, an Informed Consent Form (ICF) with total freedom to refuse to participate or to withdraw their consent at any stage of the research. Coding data guaranteed anonymity and confidentiality. The Research Ethics Committee of the University of São Paulo School of Nursing (EEUSP) approved this study.

### Design, period, and place of study

This study is quantitative, cross-sectional, and descriptive. The data were collected from August to December 2021 using a Google Forms^®^ electronic form. To understand nursing professional values, the Nurses Professional Values Scale-3 (NPVS-3) validated for Brazil^(9)^ was used. The NPVS-3 comprises 28 items with a Likert scale ranging from 1 (not important) to 5 (most important). Each item of the NPVS-3 is a short descriptive sentence reflecting a specific ethical code provision. We worded all items positively; none of them had reversed scores. The possible range of scores is 28 to 140. The higher the score, the stronger the professional value orientation. We obtained total scores by adding the numerical responses for each item. It is a self-administered scale that takes approximately 15 minutes to complete^(10)^.

Three dimensions support the NPVS-3: “Caring centered on the collective,” composed of ten questions that address the professional’s relationship and commitment to the patient, family, group, and community; “Activism,” with ten questions, which refer to the nurses’ responsibility to the community, reflecting their role in public health policies to promote comprehensive and universal access; and “Professionalism,” with eight questions, which consider the proficiency of the practice, professional growth, and responsibility for the environment.

The Caring dimension covers the items: Respect the dignity, values, and human rights of all individuals; Protect the health and safety of the patient/public; Take responsibility for one’s practice; Protect the moral and legal rights of patients; Defend the patient; Provide care without bias or partiality to patients and populations; Ensure the patient’s right to confidentiality and privacy; Confront professionals presenting questionable or inappropriate practices; Protect the rights of research participants, and Establish practices guided by principles of loyalty and respect for the person.

The items in the Activism dimension are: Promote advancement of the profession through up-to-date knowledge of health-related actions; Recognize the role of nursing professional associations in health policy making; Establish collaborative partnerships to reduce discrepancies to access health care; Take responsibility for meeting the health needs of various populations; Participate in nursing research and/or implement research findings appropriate to practice; Actively promote the health of populations; Participate in professional efforts and collaborative interactions to ensure quality care; Promote mutual peer support and collaborative interactions to ensure quality care and professional satisfaction; Act to influence legislators and other policy makers to improve health care; and Engage in consulting/collaborations to provide the best health care.

Finally, the Professionalism dimension encompasses the following items: Performing continuous self-assessment; Taking responsibility for one’s own well-being; Participating in peer evaluations; Setting standards as a guide for practice; Promoting and maintaining standards where there are learning activities for students in a planned fashion; Taking initiatives to improve practice environments; Attending courses to update knowledge and skills and thus maintain competence; and Recognizing professional boundaries.

For the elaboration of the manuscript, we were guided by the recommendations of the Strengthening the Reporting of Observational Studies in Epidemiology (STROBE), followed by the SQUIRE tool.

### Population or sample; criteria of inclusion and exclusion

The sample consist of nursing students enrolled in any semester in three Brazilian public institutions - two in the Southeast Region and one in the North Region of Brazil, one state university, and two federal universities. We chose those universities by convenience. To maintain anonymity, we coded the universities as 1 (State-Southeast Region), 2 (Federal-Southeast Region), and 3 (Federal-North Region).

The sample size calculation was made by proportion, with the following parameters: the total universe of students (p); the proportion of enrolled students (1 - p); the proportion of individuals enrolled per semester (d); Confidence level (1 - α) - No value (z). The formula considered: Acceptable precision = 5%; Degree of confidence = 95%; Actual value < 20%, Total population proportion (p) = 20%; Confidence level (1 - α) = 95% equals 1.96; and Precision (d = 15% 25%), with a sampling error of 5%, corresponding to 210 (62.3%) students from University 1; 47 (13.9%) from University 2, and 80 (23.8%) from University 3.

As inclusion criteria, we considered the student regularly enrolled in the Nursing course of the universities participating in the study, and as exclusion criteria, failing to answer more than 80% of the data collection instrument.

### Study protocol

Researchers allocated in the participating institutions sent the invitation to participate in the study by virtual dissemination (e-mail and WhatsApp), including the link to the electronic page with Informed Consent and data collection questionnaire (gender, university where the student was enrolled and the Nurses Professional Values Scale-3 [NPVS-3]).

### Analysis of results and statistics

The statistical analysis for comparison between universities was performed using the Statistical Package for the Social Sciences^®^ (SPSS), version 15.0. The Wilcoxon Mann Whitney test and the Kruskal-Wallis test by ranks (ANOVA) were employed for comparisons, and they tested whether the variables originated from the same distribution. The significance level accepted was p < 0.05.

## RESULTS

A total of 337 nursing students participated in this study, 282 female (83.68%) and 55 male (16.32%). Only 30 (8.90%) declared themselves black, and 20 (5.93%) declared themselves indigenous. University 1 showed the highest percentage of student participation (210; 62.31%), as well as students enrolled in the 6t^h^ semester (83; 24.92%) (Table 1).

**Table 1:** Distribution of nursing students according to gender, color/race, and attending university and semester, Brazil, 2021

Variables	n	%
Gender		
Female	282	83.68
Male	55	16.32
Color/Race Self-declared		
Yellow	135	40.06
White	104	30.86
Indigenous	20	5.93
Brown	48	14.24
Black	30	8.90
University		
University 1 - Southeast	210	62.31
University 2 - Southeast	47	13.95
University 3 - North	80	23.74
Semester		
1^st^	2	0.60
2^nd^	50	15.02
3^rd^	28	8.41
4^th^	58	17.42
5^th^	21	6.31
6^th^	83	24.92
7^th^	27	8.11
8^th^	58	17.42
9^th^	6	1.80
No answer	4	1.19

Statistical data from the Professional Values Scale (NPVS-3) of Brazilian nursing students lead to mean scores of 46.61, 43.78, and 34.55 for the Caring, Activism, and Professionalism dimensions, respectively (Table 2). The Caring dimension presented higher mean scores than the others in the three universities, which suggests that it is the most valued by students. The results regarding the regions and the scope of administration were the same (federal or state).

**Table 2:** Distribution of Nursing students according to the dimensions and total score of the Professional Values Scale (NPVS-3), Brazil, 2021

Dimension (N = 337)	Mean	Standard- deviation	Minimum	Median	Maximum
Caring	46.61	3.70	25	47.00	50
Activism	43.78	5.66	28	45.00	50
Professionalism	34.55	3.80	21	35.00	40
Total	124.93	11.72	86	128.00	140

A statistically significant association (p < 0.05) was found for the Caring dimension with the variables “where they attend university” and “gender,” in which higher mean scores were assigned by students from universities 2 and 1, respectively, and by female gender. The Activism dimension and total rating of professional values were also associated with gender, with higher scores attributed to female students (Table 3).

**Table 3:** Association of the dimensions and total score of the Professional Values Scale (NPVS 3) with the universities and the study participants’ gender, Brazil, 2021

Variable	n	Mean	SD^*^	Min†	Maxǂ	*p* value
Caring						
University 1	210	46.84	3.68	25	50	0.016^§^
University 2	47	47.17	2.83	38	50	
University 3	80	45.67	4.09	29	50	
Female	282	46.77	3.68	25	50	0.015^**^
Male	55	45.76	3.76	33	50	
Activism						
University 1	210	43.71	5.65	28	50	0.908^§^
University 2	47	43.89	6.13	29	50	
University 3	80	43.90	5.49	28	50	
Female	282	44.03	5.66	28	50	0.030^**^
Male	55	42.49	5.58	28	50	
Professionalism						
University 1	210	34.41	3.74	24	40	0.528^§^
University 2	47	34.89	4.37	21	40	
University 3	80	34.69	3.65	24	40	
Female	282	34.67	3.80	21	40	0.192^**^
Male	55	33.93	3.81	24	40	
Total						
University 1	210	124.96	11.58	86	140	0.610^§^
University 2	47	125.96	12.14	94	140	
University 3	80	124.26	11.93	86	140	
Female	282	125.47	11.77	86	140	0.020^**^
Male	55	122.18	11.16	88	140	

* SD - Standard deviation; †Min - Minimum; ǂMax - Maximum; §Kruskal-Wallis test;

** Wilcoxon Mann-Whitney test.

## DISCUSSION

Nursing is characterized as a female profession, evidenced by their higher participation in this study. It is a profession of care in which female attributes, such as dedication and patience, are considered essential, reaffirmed by the creation of Nursing Schools under exclusive boarding schools for girls. Even after the inclusion of the male gender in its Brazilian educational (1905) and institutional context, it is not uncommon to recruit men for jobs that require qualities described as naturally masculine, such as physical strength^(11)^. The presence of men is still very recent in some countries, such as Turkey, where its incorporation only took place in 2007 through a new nursing law, and its acceptance is proceeding gradually due to the country’s culture^(12)^. Despite the increase of men in nursing, it is still possible to affirm that the profession remains a female job^(11)^; in this circumstance, there is a significant difference in the perception of professional values between men and women in the three evaluated dimensions.

The absence of its unique code of ethics compels Turkey to use the American Nurses Association declarations (ANA), an organization that protects and promotes the nursing profession. Unlike the Brazilian public health system, they divide the organizations into public, private, and university institutions. In public institutions, expenses are paid according to the patient’s income, who must be working, retired, or a refugee; their dependents are entitled to access the system through a payment deductible from the wages of the person responsible; citizens below the poverty line have free access to services. In private institutions, users (excluding those with health insurance) pay the entire bill. In university hospitals, the government pays part of the expenses^(1,13)^. Despite the differences, it is possible to observe, in the study conducted to measure the professional values of nursing students at Uludag University in Bursa, Turkey, a similarity that revealed a higher mean score for Caring, along with lower participation of male students (27.1%)^(14)^.

Brazil adopted the Unified Health System (SUS), which has universality, integrality, and equity as its principles. However, it is taken into consideration that its population heterogeneity creates a variation of professional Nursing values in each state. That variation is because the universities’ teaching systems are regulated according to their location but share the prioritization of collective values. That is evidenced by the higher score for the Caring dimension in the three universities since the essence of the nurse is patient care, holding as competence the meeting of social health needs. Despite that, students’ understanding of the indispensability of the values of Professionalism and Activism related to the Caring dimension is of great importance for the professional influence on health reform^(15)^.

However, to try to understand the supremacy of the Caring dimension, it is necessary to look deeper into the literature of Nursing education, which has been transmitted throughout the generations of future nurses through its insertion in the curriculum grid since the first semester of graduation. Initially, they based care on the spirit of donation and abnegation, associated with religiosity, with the enforcement of three virtues: modesty, simplicity, and charity. With the establishment of Modern Nursing by Florence Nightingale, there is a transformation of the nursing concept, which serves as a basis for the current nursing, provided through humanized care assistance, a principle considered essential for patients’ recovery, based on solidarity, helpfulness, and morality^(7,16)^.

Recent social changes, however, have been impacting nursing professional values to the point that places them as a consumer good, with the appreciation of the value of productivity, highlighting differences between professional values considered essential for the care and institutional requirements^(16)^. Therefore, it is imperative to recover their values since such differences can negatively affect nurses’ professional identity and, consequently, their decision-making^(17)^.

The Activism dimension obtained an intermediate score. The item “Promote the progress of the profession through updated knowledge of health-related actions” is a factor that needs constant development, with the adoption of educational practice as a health promotion strategy. Therefore, educators should constantly carry out research and extension projects to encourage students to develop the power of evaluation and judgment. As for the “Act to influence legislators and other policymakers to improve health care,” there is still negligence due to a lack of motivation and knowledge, time limitations, and insufficient emphasis on nursing educators^(17)^. That requires the engagement of professionals and students to fight against inequalities in access to healthcare and injustices in the hierarchical hospital environment.

The Professionalism dimension provides services based on respect, commitment, and defense of patient rights, compliance with the code of ethics, and commitment to professional improvement. According to Marçal and Zagonel: “The student must form their professional identity during training to understand their role in the healthcare team and its performance with their particular body of knowledge.” As a manner to improve teaching, the development of teaching professionals is necessary to guarantee students adequate models of professionalism associated with the use of active methodologies. That is important because many institutions are still based on traditional pedagogy, in which students act passively, and the educator decides about the content, context, methods, and assessments. Professionalism assessment is a tool to improve skills and develop students’ difficulties; thus, the Professionalism dimension is not 100% achieved, not even by the last students in the last year of graduation, because they need constant improvement due to the turnover of the practice field^(18)^.

Some consistent values in the NPVS-3 correspond to the CEPE values, such as: in the Caring dimension, the item “Respect the dignity, values, and human rights of all individuals” is related to CEPE’s Article 25 (Record in the patient’s medical chart the information inherent and indispensable to the care process), whose understanding is that the exercise of empathy and respect for others is the basis for nursing practice. On the other hand, in the Professionalism dimension, “Participate in peer evaluations” obtained the lowest score, which we can explain because some students need to be made aware of the concept of peer evaluation, which highlights the importance of mutual evaluation among the nurses on the team. Moreover, in CEPE, no specific article deals with that subject.

In this context, the education students receive during each semester of the undergraduate course is essential for reinforcing the nurse’s professional identity. In this study’s findings, we observed that in the last semester, students achieved higher scores in all dimensions compared to the other semesters. It is believed that, consciously or unconsciously, university professors have worked on professional values during the structure of those students’ careers - values that are the basis for the nurses’ professional identity. Nobre et al. point out that students, professors, and nurses, when identifying possible limiting values, such as demotivation and negative emotions due to low salaries or an inharmonious work environment, can create strategies for behavioral changes during professional practice^(7)^. The same applies to identifying facilitating values, such as teamwork, planning, and organization, which can address personal and environmental stimuli, creating genuine relationships between the individual and their profession.

### Study limitations

One of the limitations of this study is the lack of assessment of cultural values and their influence on students’ professional values. Culture is multifactorial and diverse; people follow the rules and behaviors pre-established by their environment, often without questioning or self-criticism, keeping cultural feedback. Thus, evaluating the culture of those students will allow us to analyze and understand mechanisms of evolution in the Caring, Activism, and Professionalism dimensions that influence professional conduct.

### Contributions to the field of Nursing

The absence of the development of professional values and student leadership during training impacts the initial performance of the professional. Therefore, having an instrument to estimate nursing students’ professional values will help faculty analyze and implement teaching-learning strategies.

## CONCLUSIONS

Building on professional values-based learning allows students to contemplate the importance of reinforcing their professional identity and making values-based decisions.

Professional values are a compilation of usual positions and practices among individuals of a specific professional category, thus serving as beacons guiding the actions in pursuit of the objectives.

In this study, the professional values of nursing students at the participating universities are more oriented toward the Caring dimension. This result portrays the very choice of profession. Nursing is the profession of care; its history emphasizes assistance as the primary aspect of its essence. Let’s analyze the Brazilian Curricular Guidelines for the Nursing course. Almost 90% of the nurses’ training focuses on assistance learning. Understanding the deficits in developing values in other dimensions will contribute to teaching and learning efforts focused on them.

Nowadays, several types of activism are appreciated. So, mapping the professional values requires an explicit and formal declaration of the behavior and causes defended by workers of the category. In this sense, to show the professional values of nursing since the formation is to strengthen the profession’s identity, increasing the engagement of “Being a Nurse.”

## References

[1] Özsoy S, Özturk Donmez R. (2015). Nurses professional values scale-revised: psychometric properties of the Turkish version. Nurs Pract Today.

[2] Sousa LB, Barroso MGT. (2009). Reflexion on the care as essence of nursing leadership. Esc Anna Nery.

[3] Posada ASH. (2009). [Some considerations about human values and the nursing professional]. Aquichan.

[4] Donmez RO, Ozsoy S. (2016). Factors influencing development of professional values among nursing students. Pak J Med Sci.

[5] Sousa GC, Rached CDA. (2021). The influence of personal and professional values on nursing leadership: an integrative review. Int J Health Manag Rev.

[6] Guimarães GL, Chianca TC, Mendoza IY, Goveia VR, Viana LO. (2015). The core values of modern nursing in the light of Dilthey and Scheler. Texto Contexto Enferm.

[7] Nobre TC, Heliodoro EA, Rosa DO. (2021). The personal and profissional values of nurse. Enferm Foco.

[8] Conselho Federal de Enfermagem (COFEN) (2017). Resolução COFEN no 564/2017.

[9] Rached CDA, Ferreira VCG, Santos JN. (2022). Brazilian Version of the Nursing Professional Values Scale (NPVS-3): Validity and Reliability Assessment. J Nurs Meas.

[10] Weis D, Schank MJ. (2017). Development and Psychometric Evaluation of the Nurses Professional Values Scale-3. J Nurs Meas.

[11] Leal SMC, Lopes MTM. (2005). The persistent feminization in Brazil’s professional nursing education. Cad Pagu.

[12] Costa KDS, Freitas GFD, Hagopian EM (2017). Men in nursing: academic training after graduation and professional trajectory. Rev Enferm UFPE.

[13] Aydogdu ALF. (2020). Pandemic caused by the new Coronavirus: health system and coping measures in Turkey. J Nurs Health.

[14] Ayla IA, Ozyazicioglu N, Atak M, Surenler S. (2018). Determination of professional values in nursing students. Int J Caring Sci.

[15] Vijayalakshmi P, Narayanan A, Thankachan A, Changhorla A, SaiNikhil Reddy S. (2021). Professional and ethics values in nursing practice: an Indian perspective. Invest Educ Enferm.

[16] Donoso MTV, Wiggers E. (2020). Discussing the periods before and after Florence Nightingale: nursing and its historicity. Enferm Foco.

[17] Poorchangizi B, Borhani F, Abbaszadeh A, Mirzaee M, Farokhzadian J. (2019). The importance of professional values from nursing students perspective. BMC Nursing.

[18] Marçal ARV, Zagonel IPS. (2020). Professionalism in nurse’s education: apprehension of the meanings of professors and students. J Nurs Health.

